# miR-200 Enhances Mouse Breast Cancer Cell Colonization to Form Distant Metastases

**DOI:** 10.1371/journal.pone.0007181

**Published:** 2009-09-29

**Authors:** Derek M. Dykxhoorn, Yichao Wu, Huangming Xie, Fengyan Yu, Ashish Lal, Fabio Petrocca, Denis Martinvalet, Erwei Song, Bing Lim, Judy Lieberman

**Affiliations:** 1 Immune Disease Institute and Department of Pediatrics, Harvard Medical School, Boston, Massachusetts, United States of America; 2 The John T. Macdonald Foundation of Human Genetics and the Department of Microbiology and Immunology, Miami Institute for Human Genomics, University of Miami Miller School of Medicine, Miami, Florida, United States of America; 3 Stem Cell and Developmental Biology, Genome Institute of Singapore, Singapore, Singapore; 4 Department of Breast Surgery, No. 2 Affiliated Hospital, Sun-Yat-Sen University, Guangzhou, People's Republic of China; 5 Department of Medicine, Beth Israel Deaconess Medical Center, Harvard Medical School, Boston, Massachusetts, United States of America; Roswell Park Cancer Institute, United States of America

## Abstract

**Background:**

The development of metastases involves the dissociation of cells from the primary tumor to penetrate the basement membrane, invade and then exit the vasculature to seed, and colonize distant tissues. The last step, establishment of macroscopic tumors at distant sites, is the least well understood. Four isogenic mouse breast cancer cell lines (67NR, 168FARN, 4TO7, and 4T1) that differ in their ability to metastasize when implanted into the mammary fat pad are used to model the steps of metastasis. Only 4T1 forms macroscopic lung and liver metastases. Because some miRNAs are dysregulated in cancer and affect cellular transformation, tumor formation, and metastasis, we examined whether changes in miRNA expression might explain the differences in metastasis of these cells.

**Methodology/Principal Findings:**

miRNA expression was analyzed by miRNA microarray and quantitative RT–PCR in isogenic mouse breast cancer cells with distinct metastatic capabilities. 4T1 cells that form macroscopic metastases had elevated expression of miR-200 family miRNAs compared to related cells that invade distant tissues, but are unable to colonize. Moreover, over-expressing miR-200 in 4TO7 cells enabled them to metastasize to lung and liver. These findings are surprising since the miR-200 family was previously shown to promote epithelial characteristics by inhibiting the transcriptional repressor Zeb2 and thereby enhancing E-cadherin expression. We confirmed these findings in these cells. The most metastatic 4T1 cells acquired epithelial properties (high expression of E-cadherin and cytokeratin-18) compared to the less metastatic cells.

**Conclusions/Significance:**

Expression of miR-200, which promotes a mesenchymal to epithelial cell transition (MET) by inhibiting Zeb2 expression, unexpectedly enhances macroscopic metastases in mouse breast cancer cell lines. These results suggest that for some tumors, tumor colonization at metastatic sites might be enhanced by MET. Therefore the epithelial nature of a tumor does not predict metastatic outcome.

## Introduction

miRNAs are small ∼22 nucleotide RNAs that regulate gene expression post-transcriptionally in a sequence-specific manner to influence cell differentiation, survival and response to environmental cues [1], [2]. Each miRNA may regulate the expression of many target genes [3]. Dysregulation of miRNA expression has been linked to tumor development and metastasis. miRNAs can act as tumor suppressors (e. g. miR-15a and miR-16-1 [4]), oncogenes (e. g. miR-155 [5], [6] and miR-21 [7], [8], [9], [10]) and as promoters (e. g. miR-10b, miR-182 and miR-29a [11], [12], [13]) or suppressors (e. g. miR-335 and miR-126 [14]) of metastasis. The let-7 family of miRNAs is poorly expressed in human breast tumor-initiating cells (BT-IC) and increases as they differentiate. let-7 regulates self-renewal and differentiation by silencing H-RAS and HMGA2 expression, respectively [15]. Exogenous expression of let-7 reduces both primary tumor formation and metastasis of BT-IC in immunodeficient mice.

Two leading theories about the origin of metastases are the cancer stem cell hypothesis [16]–[20] and the EMT hypothesis [21], [22]. The former suggests that just as tumor-initiating cells with stem cell properties may be responsible for initiating a primary tumor, they also play an important role in initiating a metastasis. The latter suggests that in epithelial tumors, cells on the periphery of the tumor receive signals from the surrounding stroma, which confer mesenchymal properties allowing cells to detach from neighboring cells and become motile. These two competing theories may actually be complementary, since breast cancer stem cells have mesenchymal features [15], [23], [24]. The EMT plays an important role in tissue remodeling during embryonic development [25]. One of the hallmarks of EMT is loss of E-cadherin expression. E-cadherin is a Ca^2+^-dependent transmembrane glycoprotein that plays an important role in the maintenance of cell polarity by mediating cell-cell and cell-substratum adhesion. In addition, the intracellular domain of E-cadherin interacts with the cytoskeleton through its association with the catenins. The EMT involves the loss of cell-cell adherence through the repression of E-cadherin (Cdh1) expression and the rearrangement of the cytoskeleton. This causes the cells to adopt a more elongated appearance resembling fibroblasts and facilitates cellular motility, a key event in tumor invasiveness. The EMT can be triggered by the expression of various transcription factors, including the E-box binding factors Snai1 (snail) and Snai2 (slug), in response to soluble factors present in the tumor microenvironment, such as TGFβ [26], [27]. The transcriptional repressors, Zeb1 (TCF8 or δEF1) and Zeb2 (ZFXH1b or SIP1), suppress the epithelial phenotype by inhibiting E-cadherin expression. During normal development, the EMT can sometimes be reversed (referred to as the mesenchymal-to-epithelial transition (MET)) as a necessary step in tissue construction (for example, the formation of kidney epithelium from the nephric mesenchyme). Some recent studies suggest that a reversion of the mesenchymal phenotype of malignant cells may facilitate the establishment of macroscopic metastases [22], [25], [28].

The development of metastases requires that primary tumor cells acquire genetic or epigenetic variations that allow them to complete the required steps in metastasis - intravasation, survival in the circulatory system, extravasation, and survival and growth in distant tissues. Although miRNAs have been identified that regulate the formation of metastases, these studies have principally examined the early stages of metastasis and not the formation of macroscopic metastatic nodules [11], [14]. To begin to address factors that regulate the final step of metastasis (colonization of distant tissues), we took advantage of four well-characterized mouse mammary tumor cell lines derived from a single spontaneously arising, mammary tumor in a BALB/c mouse [29]. Although each of these tumor cells (67NR, 168FARN, 4TO7 and 4T1) is able to form primary tumors when implanted into the mammary gland of BALB/c mice, they have different metastatic properties. 67NR cells form primary tumors readily, but tumor cells do not intravasate. 168FARN cells can be detected in lymph nodes but rarely in other tissues, suggesting that they can enter the vasculature, but extravasate inefficiently. 4TO7 cells can disseminate from primary mammary tumors into the lungs but don't form visible lung nodules. Moreover, disseminated 4TO7 cells in the lungs rapidly disappear when the primary tumor is removed, suggesting that they are unable to colonize distant sites. 4T1 cells are fully metastatic and form macroscopic lung nodules from primary mammary tumors. Implantation of syngeneic breast tumor cells within the mammary gland of immunocompetent mice may more accurately mimic in vivo tumor formation in the context of all stromal factors than models involving xenotransplantation in immunodeficient mice.

Since 4TO7 cells can perform all of the steps in metastases except forming macroscopic metastatic nodules, we compared miRNA expression in 4TO7 cells with the highly metastatic 4T1 cells to determine if miRNAs might play a role in this important transition. Expression of some members of the miR-200 family of miRNAs (miR-429, miR-200b, and miR-200c) was increased more than 100-fold in 4T1 cells compared to 4TO7 cells. Although highly homologous, the miR-200 family members (miR-141, miR-429, miR-200a, miR-200b and miR-200c) can be divided into two functional groups based on their seed sequences, nucleotides 2 to 7 of the miRNA, which play an important role in target recognition. The 2 groups differ by a single seed nucleotide - miR-200b, miR-429 and miR-200c share the 5′-AAUACU-3′seed sequence and miR-200a and miR-141 have the 5′-AACACU-3′ seed. In addition, they are encoded from 2 gene clusters in mice - miR-200c and miR-141 on chromosome 6 and miR-200b, miR-200a and miR-429 on chromosome 4. In agreement with recent papers [30]–[37], we found that the miR-200 family members target the transcriptional repressor Zeb2. As a consequence of Zeb2 silencing, E-cadherin levels increase, leading to the adoption of a more epithelial phenotype. However, unlike the published reports, we found that Zeb2 suppression and the resulting increase in E-cadherin enhanced the metastasis of 4TO7 cells, allowing them to form macroscopic lung nodules. These results suggest that reestablishing an epithelial-like phenotype may be important for effective colonization of distant tissue sites by some tumors.

## Results

### The miR-200 family is up-regulated in 4T1 cells

**Figure 1:**
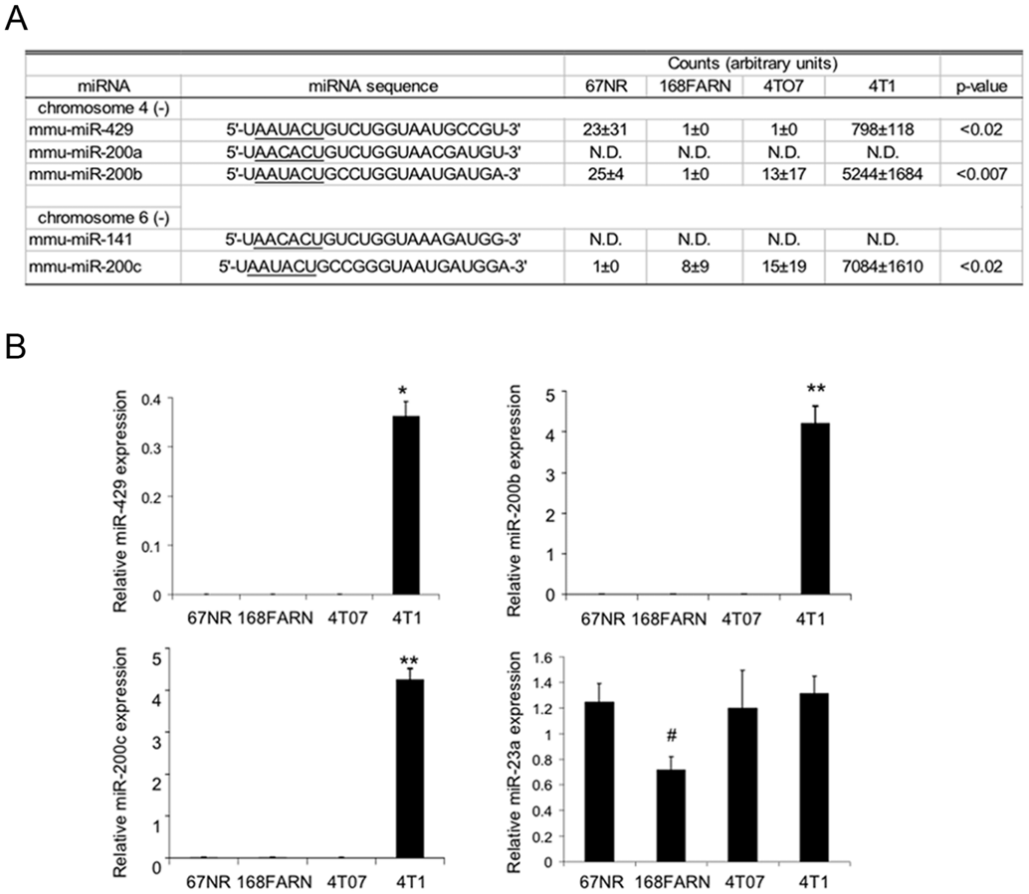
MiR-200 family member expression distinguishes highly metastatic 4T1 cells from 67NR, 168FARN, and 4TO7 cells. (A) miRNA microarray analysis of miR-200 family expression in 4 isogenic mouse breast cancer cell lines. The seed sequence (nucleotides 2–7) of the miRNA is underlined. No significant signal was detected for miR-200a and 141 (N.D. = not detected), averaged signal for all samples below 500), but the remaining miR-200 family members were highly expressed in 4T1 cells relative to the less metastatic 67NR, 168FARN, and 4TO7 cells. (B) miR-200 family expression, analyzed by qRT–PCR and normalized to U6 snRNA, confirms the microarray data. miR-23a, which is expressed in all the lines, was analyzed as a control (*, p<0.001; **, p<0.002; #, p<0.04). Data represent the mean and standard deviation from 3 independent experiments.

The most prominent change in miRNA expression by microarray analysis between the three cell lines not able to colonize distant sites (67NR, 168FARN and 4TO7 cells) and 4T1 cells was up-regulation of several members of the miR-200 family in 4T1 cells (Figure 1A, Table S1). The cells incapable of colonization had very low to undetectable expression of all miR-200 family members, while 4T1 cells expressed miR-429, miR-200b and miR-200c. These microarray results were verified by qRT-PCR (Figure 1B). Higher expression of the miR-200 family in the cell line capable of forming distant metastases was unexpected since the miR-200 family has been linked to epithelial differentiation [30]–[36] that is normally associated with decreased metastatic potential. In fact, expression of miR-429, miR-200b and miR-200c was elevated in the highly metastatic 4T1 cells but absent from 4TO7 cells, which can perform all of the steps leading up to the establishment of lung metastases except establishing the secondary tumor. Neither miR-200a nor miR-141 expression was detected in any of the cell lines, which is surprising since they would be expected to be co-expressed on the same primary transcript with the other clustered miRNAs. This suggests that either the specific probes for these miRNAs on the microarrays are not efficient at detecting these family members or that these isoforms might be differentially processed compared to the other family members, as has been recently shown to be the case for miR-181 family members [38]. The lack of miR-200a and miR-141 expression was confirmed by quantitative RT-PCR analysis (data not shown).

### The miR-200 target gene Zeb2 is down-regulated and E-cadherin is up-regulated in 4T1 cells

**Figure 2:**
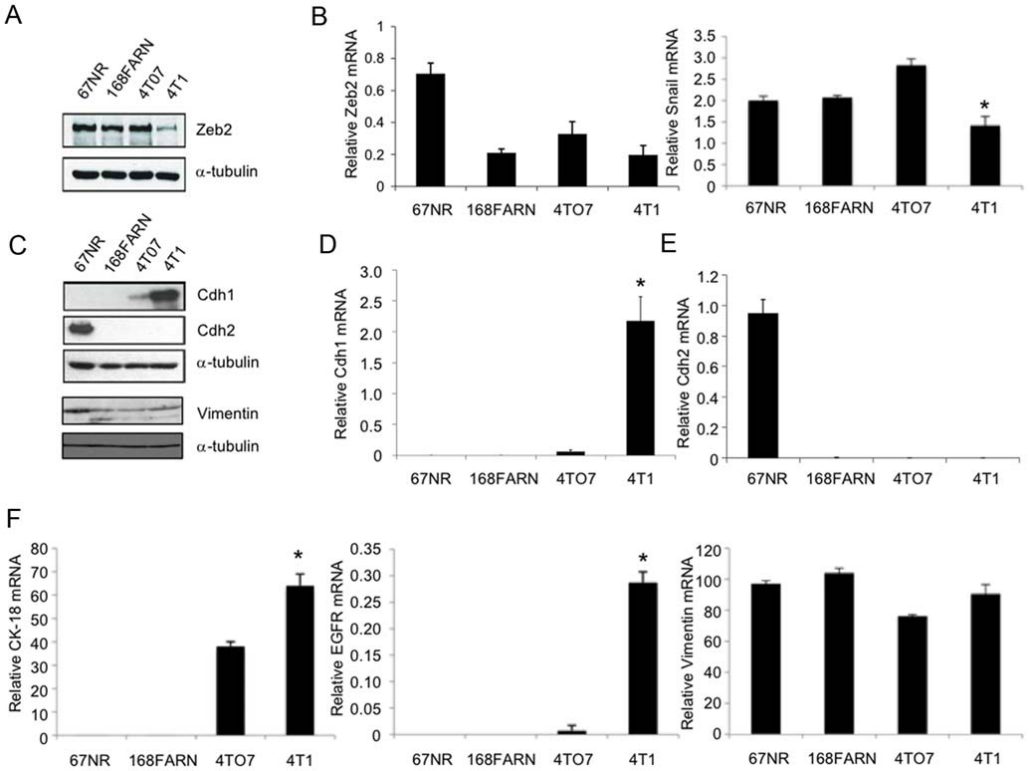
Protein expression of Zeb2 protein negatively correlates and E-cadherin positively correlates with miR-200 expression. (A) Zeb2 protein, analyzed by immunoblot relative to α-tubulin as a loading control, is significantly lower in 4T1 cells. (B) Zeb2 mRNA, analyzed by qRT-PCR and normalized to Gapdh, is higher in 67NR cells but similarly expressed in the other cell lines. Snail mRNA is somewhat lower in 4T1 cells than the other cell lines. (C–E) E-cadherin protein (C) and mRNA (D) expression is only detected in 4T1 cells, while N-cadherin protein (C) and mRNA (E) is restricted to 67NR cells. Vimentin protein (C) is expressed in all 4 cell lines, but expression is greater in 67NR cells, while vimentin mRNA is expressed at similar levels in all 4 cell lines (F). Cytokeratin-18 (CK-18) mRNA is expressed in 4TO7 and 4T1 cells, while Epidermal Growth Factor Receptor (EGFR) is limited to 4T1 cells (F). Protein was analyzed relative to α-tubulin by immunoblot and mRNA was quantified by qRT-PCR relative to Gapdh. Levels of protein and mRNA for both cadherins changed in parallel. The qRT-PCR results represent the mean and standard deviation from three independent experiments (*p<0.01, **p<0.001).

The TargetScan5.0 algorithm identified the zinc finger E-box binding homeobox 2 (Zeb2/SIP1/ZFXH1B) gene as the highest likelihood target gene of the miR-200 family with 6 potential miR-429/miR-200b/miR-200c binding sites and an additional 3 potential miR-141/miR-200a binding sites in its 3′UTR. Zeb2 protein was strongly expressed in 67NR, 168FARN and 4TO7 cells, but suppressed in 4T1 cells (Figure 2A). Zeb2 mRNA levels were significantly higher in 67NR cells than in the other cell lines, which had similar levels (Figure 2B). The low expression of Zeb2 protein in 4T1 cells relative to 168FARN and 4TO7 cells is consistent with inhibition of Zeb2 translation by miR-200. However, other post-transcriptional mechanisms (including other miRNAs) might explain the lack of difference in Zeb2 protein between 67NR and 168FARN and 4TO7 cells. Consistent with the known repressive role of Zeb2 on E-cadherin transcription, 4T1 cells, which have low endogenous levels of Zeb2, have high E-cadherin mRNA and protein (Figure 2C and 2D). Surprisingly, N-cadherin (Cdh2) mRNA and protein, a mesenchymal marker often reciprocally expressed with E-cadherin, was only detected in non-metastatic 67NR cells (Figure 2C and 2E). Immunoblot analysis showed that vimentin was most highly expressed in 67NR cells, but was comparable in the other 3 cell lines (Figure 2C). Vimentin mRNA was similar in all 4 cell lines. Expression of the epithelial cell-associated intermediate filament cytokeratin 18 (CK-18) mRNA was limited to 4TO7 and 4T1 cells and was higher in 4T1 cells [39] (Figure 2F). In addition, expression of Epidermal Growth Factor Receptor (EGFR) mRNA was restricted to 4T1 cells (Figure 2F). These data suggest that contrary to the EMT hypothesis, the nonmetastatic 67NR cells have a mesenchymal phenotype, while the metastatic cell lines have features of both mesenchymal and epithelial cells. Paradoxically, the most metastatic 4T1 cells have more epithelial characteristics based on enhanced Cdh1, CK-18 and EGFR expression, than the less metastatic cells.

### miR-200 over-expression in 4TO7 cells reduces Zeb2 and Snai1 and increases E-cadherin expression

**Figure 3:**
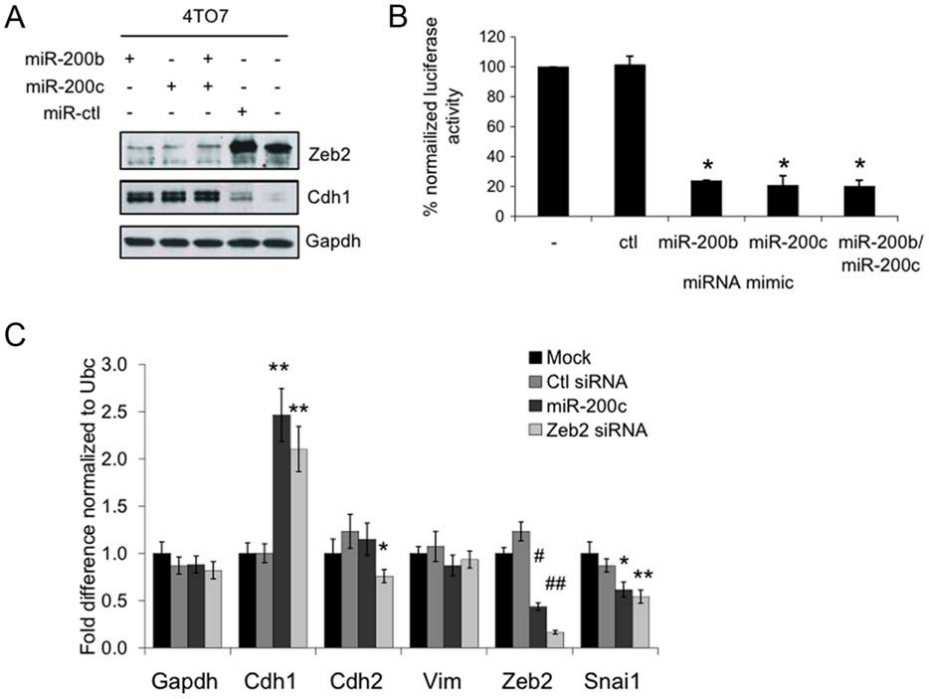
Over-expression of miR-200 in 4TO7 cells down-regulates Zeb2 expression, resulting in increased E-cadherin. (A) Zeb2 expression decreases and E-Cadherin (Cdh1) expression increases, analyzed by immunoblot relative to Gapdh, after transfection of 4TO7 cells with miR-200b and/or miR-200c. (B) miR-200 regulates the Zeb2 3′-UTR in a luciferase reporter assay. 4TO7 cells were co-transfected with the Zeb2 3′-UTR luciferase plasmid or a control vector and either the control (ctl), miR-200b and/or miR-200c miRNA mimics. The mean and standard deviation of the relative luciferase activity, normalized relative to that of mock-transfected cells, from 3 independent experiments is shown (*p<0.0002). (C) Comparison of mRNA levels of epithelial and mesenchymal markers (Cdh1, Cdh2, vimentin (Vim), Zeb2, and Snai1) in 4TO7 cells transfected with miR-200c mimic or an siRNA targeting Zeb2 (Zeb2 siRNA) or control (ctl) siRNA, analyzed by qRT–PCR normalized to the housekeeping gene Ubc. miR-200c expression increased Cdh1 mRNA, an epithelial marker, and decreased Snai1 and Zeb2, mesenchymal markers, but had no effect on N-cadherin (cdh2) or vimentin (Vim). Knocking down Zeb2 had a similar effect as expressing miR-200c, except that Cdh2 mRNA was also significantly suppressed by reducing Zeb2. Mean and standard deviation for 3 independent experiments are shown relative to the results for mock-transfected cells (*,p<0.05, **,p<0.01, ***,p<0.005, #,p<0.001 and ##,p<0.0001).

To determine whether miR-200 regulates Zeb2 and E-cadherin expression in these breast cancer cell lines, we transfected 4TO7 cells with mimics of miR-200b or miR-200c alone or in combination. Over-expressing either miR-200b or miR-200c or both led to a loss of Zeb2 expression and a concomitant increase in E-cadherin (Cdh1) levels (Figure 3A). To test the direct targeting of Zeb2 by miR-200, the complete Zeb2 3′-UTR was cloned downstream of a Renilla luciferase reporter gene. Co-transfection of the reporter plasmid with miR-200b and/or 200c in the 4TO7 cells significantly reduced luciferase expression (∼5-fold, p<0.0002), confirming previous reports [30], [32], [35] that these miRNAs suppress Zeb2 expression by recognizing sites in its 3′-UTR (Figure 3B). Transfection of both miR-200b and miR-200c had no added effect, presumably because these miRNAs redundantly bind to the same miRNA recognition sites (MRE). (Although Zeb1 is not expressed in any of the 4 cell lines under study (data not shown), the Zeb1 3′-UTR was also regulated in 4TO7 cells by miR-200b and miR-200c by luciferase assay (Figure S1).) The expression of several genes involved in determining the epithelial or mesenchymal nature of cells were also analyzed by qRT-PCR in 4TO7 cells which had been treated with the miR-200c mimic, an siRNA against Zeb2 or a control siRNA (Figure 3C). Zeb2 mRNA was significantly decreased in 4TO7 cells treated with either the Zeb2 siRNA or the miR-200c miRNA mimic. Conversely, E-cadherin mRNA increased in cells transfected with either Zeb2 siRNA (2.1-fold) or miR-200c mimic (2.5-fold). Transcripts for vimentin and N-cadherin, markers of mesenchymal cells, were not significantly altered by the miR-200c mimic, although N-cadherin mRNA was slightly, but significantly, decreased in the Zeb2 siRNA-treated cells. In addition, mRNA for the mesenchymal transcription factor Snai1 was significantly reduced in 4TO7 cells transfected with either Zeb2 siRNA or miR-200c mimic. Unlike Zeb2, Snai1 is not a predicted target of the miR-200 family. The decrease in Snai1 mRNA after treatment with miR-200c could be secondary to Zeb2 silencing and/or to recognition of a noncanonical MRE in Snai1.

### Exogenous miR-200 expression enhances the epithelial morphology of 4TO7 cells

**Figure 4:**
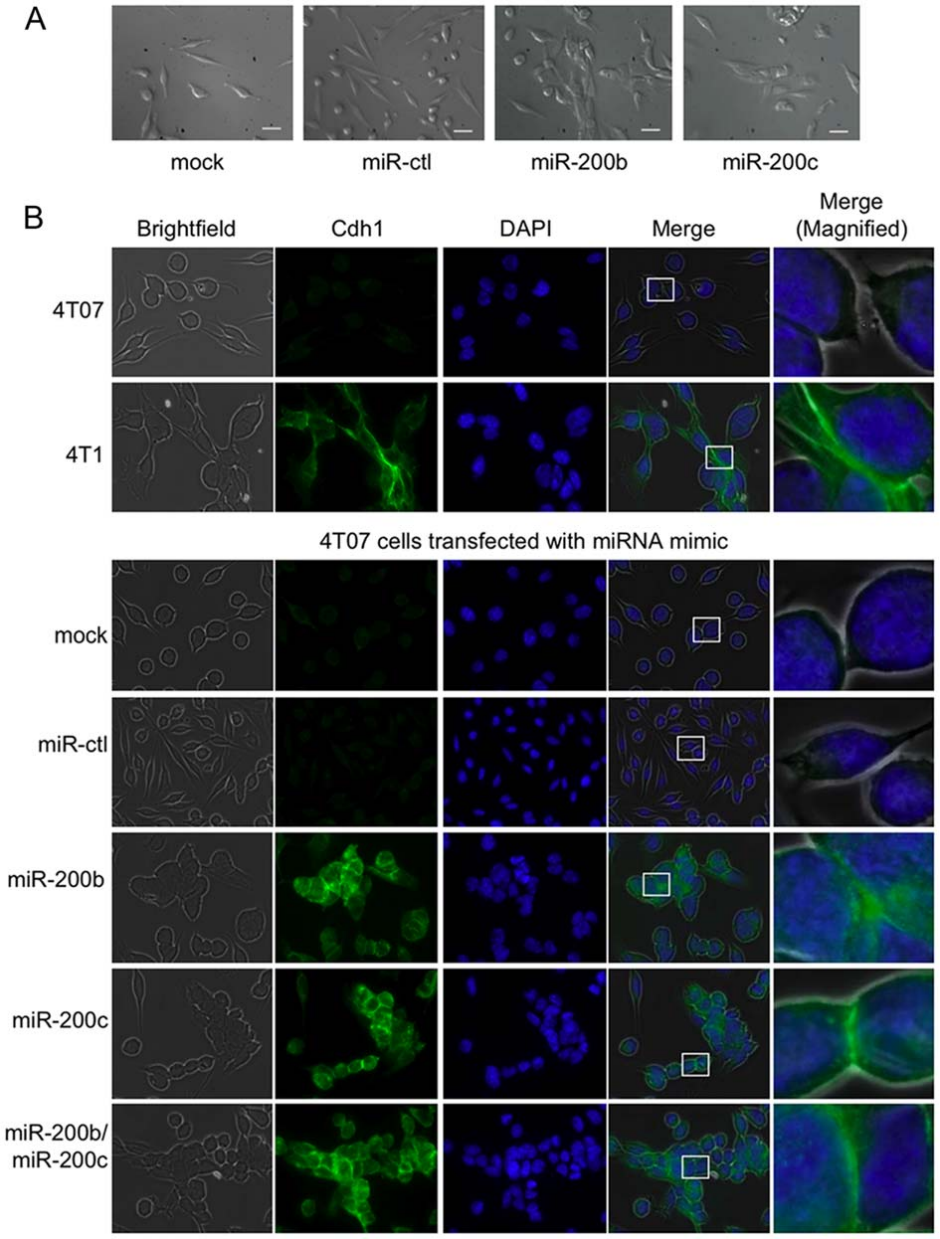
Over-expression of miR-200 in 4TO7 cells converts fibroblastic cells to an epithelial morphology. (A) Phase contrast microscopy of 4TO7 cells that were either mock treated or transfected with the miRNA control (ctl), miR-200b, or miR-200c mimic. The white bar represents 10 µm. (B) E-cadherin expression visualized by fluorescence microscopy of parental 4T1 and 4TO7 cells (top) and 4TO7 cells that were transfected with miR-200b and/or miR-200c or control mimics (bottom). 4TO7 cells treated with either of the miR-200 mimics adopted an epithelial-like morphology and expressed high levels of E-cadherin, similar to the highly metastatic 4T1 cells. E-cadherin (Cdh1) concentration at the cell-cell junction is shown in the magnified image. Cell nuclei were stained with DAPI.

The effect of exogenous miR-200 expression on E-cadherin expression and cell morphology of 4TO7 cells was also analyzed by fluorescence microscopy (Figure 4). In support of the immunoblot and qRT-PCR data, E-cadherin was readily detected in 4T1 cells, but not in 4TO7 cells. In line with this, 4TO7 cells looked more like fibroblasts growing as single cells than cultured 4T1 cells, which had epithelial morphology and adhered to their neighbors. E-cadherin in 4T1 cells concentrated at intercellular junctions. Transfection of miR-200b and/or miR-200c in 4TO7 cells increased E-cadherin expression, which also concentrated at cell junctions and shifted 4TO7 morphology from spindle-shaped cells to cobblestone-forming epithelial cells.

**Figure 5:**
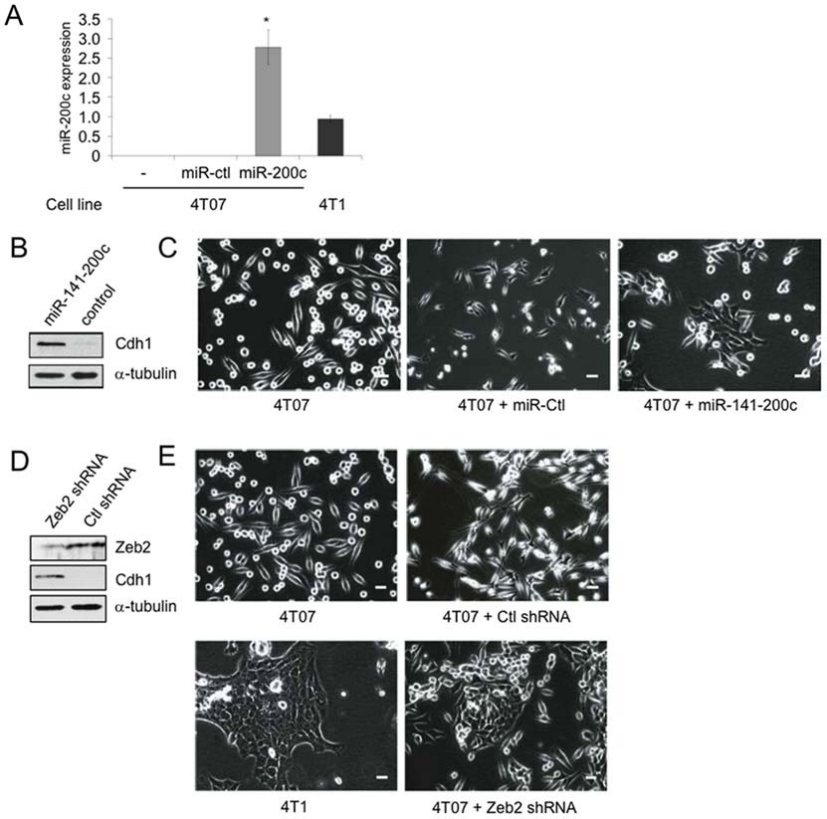
Stable miR-141-200c over-expression or Zeb2 silencing in 4TO7 cells promoted adoption of an epithelial-like morphology. (A) miR-200c expression is increased in 4TO7 cells stably expressing the miR-141-200c cluster from a retroviral vector. miR-200c levels, normalized to U6 snRNA expression, are shown relative to expression in 4T1 cells. Mean and standard deviation of 3 independent experiments are shown (*, p = 0.02, relative to 4T1 cells). (B) Stable over-expression of the miR-141-200c cluster leads to an increase in E-cadherin expression, as measured by immunoblot relative to α-tubulin. (C) The stable miR-141-200c-expressing 4TO7 cells adopt an epithelial morphology compared to the control transfected cells or the parental cells when examined by phase contrast microscopy. 4TO7 cells stably expressing a Zeb2 shRNA had reduced Zeb2 and concomitantly increased E-cadherin protein by immunoblot (D), and altered morphology from a fibroblastic to an epithelial morphology (E). 4T1 cells are shown for comparison. The white bars represent 10 µm.

To evaluate the effect of miR-200 and Zeb2 on tumor formation and metastasis, we next engineered retroviruses encoding the miR-141-200c cluster mature miRNAs or control virus expressing firefly luciferase shRNA or Zeb2 shRNA within the miR-30 stem. Infection with these retroviruses allowed us to produce 4TO7 cell lines that stably expressed the miR141-200c cluster or had stably knocked-down Zeb2. Stable expression of the miR-200c cluster in 4TO7 cells increased miR-200c expression to ∼3-fold higher than that of 4T1 cells as measured by qRT-PCR (Figure 5A). As expected, the miR-200c cluster-transfected cells expressed E-cadherin protein, which was undetected in the control virus-treated cells (Figure 5B). They also adopted an epithelial-like morphology (Figure 5C). This same phenotype was recapitulated in 4TO7 cells that had stably silenced Zeb2 expression (Figure 5D and 5E)). 4TO7 cells over-expressing miR-200 or knocked down for Zeb2 morphologically resembled 4T1 cells.

### miR-200 expression does not alter colony formation or cell proliferation, but enhances cell motility in vitro

**Figure 6:**
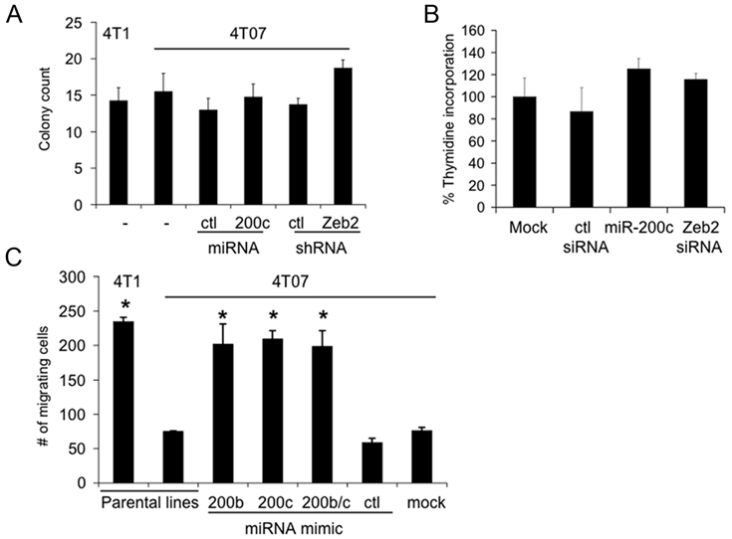
miR-200 enhances 4TO7 cells migration through a basement membrane, but does not affect cell proliferation. (A) Exogenous expression of the miR-141-200c cluster (compared to the control vector expressing luciferase shRNA) or Zeb2 shRNA (compared to a luciferase shRNA construct) in 4TO7 cells does not alter colony growth in soft agar. Parental 4TO7 and 4T1 cells also are equally efficient at forming colonies. (B) Cell proliferation as measured by thymidine incorporation is also unaffected in 4TO7 cells expressing miR-200c mimic or Zeb2 siRNA. The mean and standard deviation of 6 independent experiments is shown. (C) Transient over-expression of miR-200b and/or miR-200c enhances the migration of the 4TO7 cells through a basement membrane to the same level as the highly invasive 4T1 cells. The mean and standard deviation from analyzing five fields from triplicate samples is shown (*, p<0.01, compared to untreated (parental) 4TO7 cells).

One measure of the tumorigenic nature of cells is the ability to form colonies in soft agar. To determine the effect of miR-200c cluster expression on colony formation, soft agar assays were performed using 4TO7 cells that were untreated or stably expressed a control vector, the miR-141-200c cluster, or a Zeb2 shRNA. Altering miR-200 or Zeb2 expression did not significantly change the number or size of colonies (Figure 6A, data not shown). Consistent with these results, transient transfection of 4TO7 cells with either the miR-200c mimic or a Zeb2 siRNA (Figure 3C) did not significantly alter the rate of cell proliferation assayed by thymidine incorporation (Figure 6B).

We next performed transwell migration assays to determine the effect of miR-200b and miR-200c expression on the ability of 4TO7 cells to penetrate through an 8-µm porous membrane overlaid with basement membrane components (laminin, collagen IV, heparan sulfate proteoglycans, entactin/nidogen). Consistent with their enhanced metastatic potential, ∼3 times as many 4T1 cells migrated across the transwell as unmanipulated 4TO7 cells (Figure 6C). Transient over-expression of miR-200b and/or miR-200c in 4TO7 cells increased the number of migrating cells to that of 4T1 cells. Mock transfection or transfection of 4TO7 cells with control miRNA mimic had no effect on migration. Therefore, enhanced epithelial characteristics induced by miR-200 mimics did not impair, but rather enhanced, the in vitro migration of 4TO7 cells.

### miR-200 expression in 4TO7 cells confers in vivo metastatic potential

**Figure 7:**
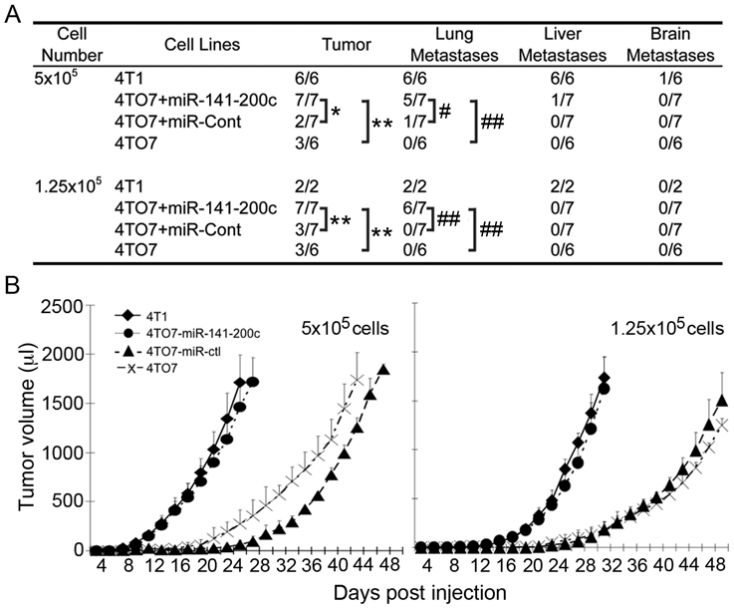
4TO7 cells stably over-expressing miR-141-200c form tumors more rapidly that are more likely to metastasize. (A) Primary tumor and metastases is enhanced in 4TO7 cells stably over-expressing the miR-141-200c cluster. The indicated number of cells was injected in the mammary fat pad of female mice (*,p = 0.02; **, p = 0.07; #, p = 0.1; ##, p = 0.005). (B) Growth curves for primary tumors in mice injected with either 5×10^5^ (left) or 1.25×10^5^ cells (right).

Since the most relevant test of metastatic potential is formation of metastases in vivo, we next assessed tumor growth and metastases after 2 doses (1.25×10^5^ and 5×10^5^ cells) of stably transduced 4TO7 cells or parental 4TO7 and 4T1 cells were implanted into the mammary fat pads of BALB/c mice (Figure 7A and 7B). For both cell doses, all mice injected with 4T1 cells formed primary tumors, but only half of the mice injected with these numbers of 4TO7 cells formed tumors. Moreover, 4T1 cells formed palpable tumors at both doses more rapidly than 4TO7 cells. While 5×10^5^ implanted 4T1 cells reached 1 ml tumor volume in ∼22 d, the same number of 4TO7 cells took almost twice as long to reach the same size in those mice which grew tumors. Although there was a longer lag time before the 4TO7 cells became palpable, once they could be detected their doubling time was indistinguishable from that of 4T1 cells at both cell doses. 4TO7 cells infected with the control retrovirus grew out comparable numbers of tumors and with the same kinetics as unmodified 4TO7 cells, while 4TO7 cells stably expressing miR-141-200c grew tumors like 4T1 cells.

**Figure 8:**
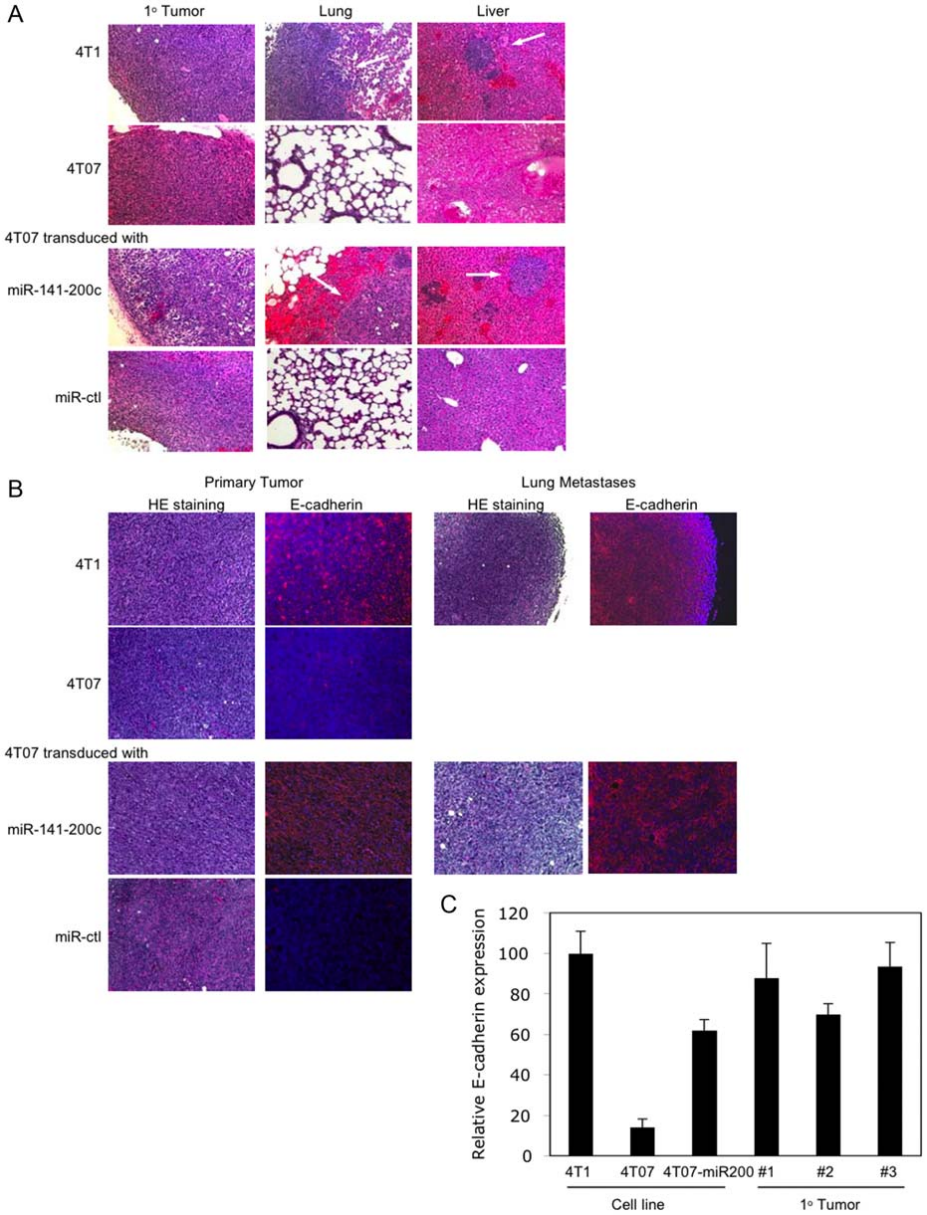
Primary tumors and metastases of 4TO7 cells stably expressing the miR-141-200c cluster express E-cadherin. (A) Representative primary mammary tumors and lung and liver tissue sections stained with hematoxylin and eosin from mice implanted with parental 4T1 and 4TO7 cells or 4TO7 cells stably-expressing miR-141-200c or control vector. 4TO7 cells and 4TO7 cells expressing the control miRNA (miR-ctl) did not form metastases. (B) Primary tumors and lung metastases formed from 4T1 cells and 4TO7 cells transduced with miR-141-200c retrovirus continue to express E-cadherin, while primary tumors from 4TO7 parental cells or 4TO7 cells infected with a control retrovirus remain E-cadherin-. (C) Primary tumors from 3 mice implanted with 4TO7 cells stably expressing miR-141-200c showed high levels of E-cadherin mRNA, analyzed by qRT-PCR and normalized to Gapdh, compared to parental 4TO7 cells. Mean and standard deviation from triplicate samples of each cell line or tumor are shown.

Mice were sacrificed when the primary tumor exceeded 15 mm diameter or when the mouse became moribund or 50 d post implantation for mice that didn't develop tumors. At the time of sacrifice, lung, liver and brain were harvested and tissue sections were stained with hematoxylin and eosin (HE) and analyzed for metastases (Figure 7A, Figure 8A). Metastases were confirmed by immunohistochemistry staining for proliferating cell nuclear antigen (PCNA) (Figure S2). All mice injected with 4T1 cells developed both lung and liver metastases. No brain metastases were detected in any mice injected with these numbers of cells. On the other hand, none of the mice injected with 4TO7 cells had evident metastases. Similarly, only 1 of 7 mice that received the higher dose of cells transduced with the control retrovirus had a lung metastasis. In contrast, most mice that received 4TO7 cells stably expressing the miR-141-200c cluster had lung metastases (5/7 at the higher dose and 6/7 at the lower dose). Therefore, 4TO7 tumors stably expressing the miR-141-200c cluster behaved like the more malignant 4T1 cells in vivo.

Because the association of epithelial characteristics with enhanced metastases was unexpected, we wanted to verify that E-cadherin continued to be expressed in transduced tumors in vivo. The primary tumors and lung metastases derived from 4T1 and 4TO7 cells transduced to express miR-141-200c stained strongly for E-cadherin, while no significant E-cadherin staining was detected in the 4TO7 tumors derived from cells that were either unmodified or infected with the control retrovirus (Figure 8B). qRT-PCR analysis also confirmed that elevated E-cadherin mRNA levels were maintained in the primary tumors derived from the miR-141-200c transduced 4TO7 cells (Figure 8C).

## Discussion

To begin to understand the molecular mechanisms that regulate the last step of metastasis - the ability to colonize distant tissues to form macroscopic metastases, we compared the miRNA profile of the isogenic, mouse mammary tumor cell lines, 4TO7 and 4T1. Although both of these cell lines can form primary tumors, intravasate, survive in the circulation and extravasate into the lungs, only the 4T1 cells form macroscopic metastases. The members of the miR-200 family of miRNAs (miR-200b, miR-200c and miR-429) were highly expressed in 4T1 cells but undetectable in 4TO7 cells. These miRNA were shown to target the transcriptional regulator, Zeb2. Zeb2, a master regulator of the mesenchymal state, exerts its effect by binding to E-box elements upstream of many genes, including E-cadherin, and repressing their transcription. As a consequence of miR-200 expression, 4T1 cells have reduced Zeb2 expression and high E-cadherin expression compared to 4TO7 cells. Consistent with this effect, 4T1 cells have an epithelial morphology with extensive cell-cell contacts while the 4TO7 cells looked more fibroblastic. Over-expression of miR-200b and/or miR-200c in 4TO7 cells, either by transient transfection of miRNA mimics or infection with a miR-141-200c cluster-expressing retrovirus, led to a loss of Zeb2 expression, an increase in E-cadherin expression and the acquisition of an epithelial-like morphology. This miRNA-mediated silencing of Zeb2 was the result of the directed targeting of the Zeb2 3′UTR by the miR-200 family, as previously reported [30]–[37]. These studies also showed that miR-200 expression enforces an epithelial phenotype in tumor cells, as we confirmed here in these mouse breast cancer cells. Based on the EMT hypothesis of cancer metastasis, it is expected that miR-200 expression would lead to a decrease in metastasis. Surprisingly, our results with this series of isogenic mouse mammary tumor cells showed the opposite effect - expression of miR-200 family members either endogenously in 4T1 cells or by retroviral transduction in 4TO7 cells enhanced both in vitro motility and in vivo metastases. In fact, in the 4 related cell lines that arose from the same spontaneously arising cancer cell clone, mesenchymal gene expression (N-cadherin, vimentin) was greatest in the nonmetastatic cell line (67NR) and decreased with increased metastatic potential, while epithelial gene expression of E-cadherin, CK-18 and EGFR followed the opposite trend. However, the 4T1 and miR-200-expressing 4TO7 cells retained some vimentin expression, suggesting that they may have some characteristics of both epithelial and mesenchymal cells.

Our results suggest that, at least in some tumors, epithelial properties may enhance the ability of tumor cells to colonize both primary and metastatic tissue sites. 4T1 cells and miR-200-expressing 4TO7 cells did not differ from parental 4TO7 cells in proliferative rate in vitro or growth in soft agar, but they both established primary tumors more rapidly and were capable of colonizing distant tissues. Although there was a delay in detecting 4TO7 primary tumors relative to 4T1 or miR-200-expressing 4TO7 tumors, once tumors became palpable, mathematical modeling did not show any significant change in their doubling times (data not shown). This suggests that epithelial characteristics enhanced colonization, but did not offer a growth advantage once a tumor had established itself.

Although the hypothesis that EMT is an important feature of metastasis has many compelling features and experimental support, other data suggest that metastasis by some tumors can be governed by processes other than EMT. The morphology and E-cadherin expression of metastatic tumors generally resembles that of the primary tumor. This has been interpreted as a potential requirement for EMT to accomplish the first steps of metastasis and a reversion (MET) to accomplish the final step of colonization. However, in this breast cancer model, the epithelial-like 4T1 cells could efficiently accomplish all the steps of metastasis, including the early steps. In fact, the more epithelial 4T1 and miR-200-expressing 4TO7 cells were better able to traverse an artificial basement membrane in vitro than their more mesenchymal relatives. However, the epithelial/mesenchymal properties of a tumor cell in vivo are not fixed. Properties of some tumor cells, particularly those at the periphery of the cancer that are closely associated with the tumor stroma, are plastic and can be modulated by response to stromal cytokines or binding to stromal receptor ligands. We cannot rule out that some of the 4T1 or miR-200-expressing 4TO7 cells transiently became E-cadherin- and more mesenchymal in vivo under the influence of local stromal factors. However, the more economical explanation of our surprising findings is that a complete EMT is not required for any of the metastatic steps in this tumor model. It may well be that these transformed cells are hybrid cells with some mesenchymal properties, as evidenced by their expression of vimentin mRNA and protein, that confer motility and invasive capability.

Although an inverse correlation between E-cadherin and invasiveness has been found for many cancers, abundant examples are inconsistent or contrary to this general assumption. Immunohistochemical analysis of mammary carcinoma expression of three epithelial markers, E-cadherin, α-catenin and β-catenin, found that they were expressed in the majority of both organ-confined intraductal breast carcinomas (100%) and invasive ductal carcinomas (∼70%) [40]. In addition, no correlation between metastatic status and epithelial marker expression was seen when examining lymph-node positive and lymph-node negative tumors [40]. Several groups have found that E-cadherin status of breast carcinomas showed weak or no correlation with vascular invasion [34], nodal status [41], [42], the presence of metastases [34], [41], and disease recurrence or survival [34], [41], [42]. Similarly, E-cadherin expression and desmosomal structure was maintained in invasive colon carcinomas and their metastases [43]. Retrospective analysis of gastric carcinomas by immunohistochemistry showed no correlation between E-cadherin status and the depth of invasion, lymph node status and level of vascular invasion in gastric carcinomas [44].

A correlation between enhanced epithelial properties and tumor metastasis similar to what we found here was previously reported using in vivo passaged transitional bladder carcinoma, TSU-Pr1 (T24) cells [45], [46]. Isogenic cell lines were derived from metastatic bone tumors that arose in immunocompromised mice following intracardiac inoculation. Although the cell lines developed from the 1^st^ (TSU-Pr1-B1) and 2^nd^ (TSU-Pr1-B2) rounds of passaging had enhanced metastatic potential compared to the parental cells, they both showed enhanced epithelial characteristics, including increased cell-cell adhesion, a decrease in vimentin and up-regulation of epithelial intermediate cytokeratin filaments [45], [46]. Moreover, reversion of epithelial characteristics to a mesenchymal morphology (spindle-like fibroblastic cell shape) by silencing the fibroblastic growth factor receptor IIIc subtype (FGFR2IIIc) impaired the development of metastases and increased mouse survival [46]. These results underlie the importance of cell-cell and cell-substratum adhesion in the development of micrometastases into macroscopic secondary tumors. The parental TSU-Pr1 cells, like the 4TO7 cells used in this study, were able to intravasate, survive in the circulatory system, extravasate and form micrometastases in the lung but were defective in forming macroscopic metastases. In each of these cases, the acquisition of epithelial characteristics enhanced the metastatic potential of the cells allowing for the transition from micrometastases to overt secondary tumors.

Several miRNAs have been implicated in the regulation of early steps of breast cancer metastasis (reviewed in [47]). miR-10b promoted metastatic transformation of breast cancer cells, while miR-373 and miR-520c enhanced extravasation and metastatic development [11], [48]. miR-21 has also been shown to inhibit the expression of multiple metastasis suppressors [49], [50]. miR-29a has also been shown to promote mesenchymal properties and promote metastasis of Ras-transformed mouse mammary epithelial cells [13]. On the other hand, miR-335 and miR-126 inhibited metastatic development [14]. In our study, some of these miRNAs (miR-10b, miR-373, miR-520c and miR-29a) were not probed in our microarrays and others (miR-21, miR-126 and miR-335) showed no significant differences in expression amongst the 4 isogenic cell lines. There was a steady increase in miR-182 expression between the 67NR cells and the 4T1 cells with a 3.8-fold increase overall. These findings suggest that alterations in different miRNAs can regulate metastasis in different tumors. It is likely that multiple combinations of transforming mutations and alterations in miRNA expression can contribute to a metastatic phenotype. In particular, it would be worthwhile to examine whether the miR-200 family might have a role in regulating the metastasis of different human breast cancer subtypes.

Although this paper is the first to show the direct enhancement of metastasis by the miR-200 family, changes in miR-200 family levels have been associated with enhanced tumorigenesis. Croce and colleagues found that the miR-200 family (miR-200a, miR-200b, miR-200c and miR-141) were upregulated in human ovarian cancers compared to normal ovarian tissue [51]. This up-regulation of miR-200 family members was particularly pronounced in serous and endometroid histotypes. A subsequent study confirmed this result in serous ovarian cancers [52]. Moreover, over-expression of the miR-200 family significantly correlated with decreased survival. Patients with ovarian tumors with high miR-200a expression had an approximately 50% decrease in median survival time compared to those lacking significant miR-200a expression (27.5 months vs. 61 months, respectively [52]). The region on chromosome 1 from which the human miR-200a-429 cluster is encoded was also found to be amplified in several epithelial cancers - ovarian cancer, breast cancer and melanoma [53]. This same region was found to undergo chromosomal gain in serous ovarian carcinoma samples when examined by high-resolution array comparative genomic hybridization [53], [54]. In addition, members of the miR-200 family of miRNAs, miR-141 and miR-200b, were found to be over-expressed in malignant cholangiocarcinoma cells compared to non-malignant cells [55]. The chromosomal locus (12p13.31) from which miR-141 and miR-200c are encoded is associated with chromosomal gain in biliary tract cancers. Over-expression of miR-141 or miR-200b is associated with an increase in proliferation, while inhibition of these miRNAs impairs cell proliferation. In addition, over-expression of miR-141 and miR-200b correlates with resistance to gemcitabine treatment [55].

The formation of metastases requires the acquisition of genetic or epigenetic changes that allow for the detachment of cells from the primary tumor, transport and survival in the circulation and the establishment of macroscopic tumors in distant organs. Given the heterogeneity of oncogenic changes associated with different tumor subtypes, it is likely that more than one pathway can promote metastases. It is clear from this work and that of others that miRNAs play an important role in regulating metastasis, the ultimate cause of death of most cancer patients. Approaches that alter miRNA expression levels, either inhibiting miRNAs that promote the development of metastases or mimicking miRNAs that promote differentiation and apoptosis, may prove to be therapeutically beneficial.

## Materials and Methods

### Cell culture

67NR, 168FARN, 4TO7 and 4T1 cells, a kind gift of Fred Miller (Wayne State University) were grown in complete medium, DMEM (Gibco) containing 10% fetal bovine serum (FBS) supplemented with 1 mM L-glutamine, penicillin/streptomycin and non-essential amino acids (Gibco).

### miRNA microarray and data analysis

Total RNA was extracted using Trizol Reagent (Invitrogen) and size fractionated using an YM-100 Microcon filter (Millipore). The isolated small RNAs (<300 nt) were 3′-extended using poly(A) polymerase and labeled with Cy5. Hybridizations were performed on a microfluidic biochip platform (LC Sciences) using 100 µL 6xSSPE buffer (0.90 M NaCl, 60 mM Na_2_HPO_4_, 6 mM EDTA, pH 6.8) containing 25% formamide at 34°C overnight. Hybridization images were collected on a laser scanner (GenePix 4000B, Molecular Device) and digitized using Array-Pro image analysis software (Media Cybernetics) with a scan resolution of 10 microns and PTM between 480 and 540V. miRNA microarrays corresponding to miRbase v9.0 (nine probes for each miRNA on one chip) were used to compare the expression of 375 miRNAs. Data were analyzed by background subtraction using a regression-based background mapping method and normalization The regression was performed on 5% to 25% of the lowest intensity data points excluding blank spots. Raw data matrix was then subtracted from the background matrix. Inter-array normalization was carried out using a cyclic LOWESS (Locally-weighted Regression) method. Normalized signals of all probes corresponding to one miRNA were averaged for individual sample and shown in the Table S1. All the miRNA microarray data is MIAME compliant and both the raw and normalized data has been deposited in the ArrayExpress database (accession number E-MEXP-2289).

### mRNA and miRNA quantification

Total RNA was prepared using Trizol Reagent (Invitrogen) and reverse transcribed using random hexamers and Superscript III reverse transcriptase. Quantitative real time PCR (qRT-PCR) was performed in triplicate samples using platinum Taq polymerase (Invitrogen) according to the manufacturer's protocol with Syber green detection using the BioRad iCycler. Results were normalized to Gapdh or Ubc as indicated. All RT-PCR primers are listed in Table S2. miRNA levels were quantified using TaqMan miRNA Assay Kit, TaqMan miRNA Reverse Transcription Kit and the Taqman 2X Universal PCR Master Mix, No AmpErase UNG (Applied Biosystems) as per the manufacturer's protocol. All values were normalized to U6 snRNA (Applied Biosystems).

### TargetScan analysis

To determine whether a gene was also a predicted target of miR-200b and c, the presence of miR-200 family binding sites was analyzed using TargetScan 5.0 (www.targetscan.org [56]).

### siRNA and miRNA mimic transfection

4TO7 cells were transfected with miRNA mimics (miR-200b and/or miR-200c) (Dharmacon) or Zeb2 or firefly luciferase siRNAs using Lipofectamine 2000 (Invitrogen). Briefly, 6×10^5^ cells were plated/well in a 6-well plate the day prior to transfection. Prior to transfection, medium was aspirated and replaced with OptiMEM (Gibco). Lipid complexes, formed according to the manufacturer's protocol, were incubated with the cells for 4 h before culture supernatants were aspirated and replaced with complete growth medium. Cells were harvested 72 h post transfection for mRNA and protein analysis. The sequences of the sense and antisense strands of the siRNAs [57] are found in Table S2.

### Luciferase assay

4TO7 cells were co-transfected with 100 nM miRNA mimics and 0.5 µg psiCHECK2 vector (Promega) encoding the 3′-UTR of Zeb2 or Zeb1 downstream of the Renilla luciferase gene using Lipofectamine 2000 as above. Cells were lysed 24 h post transfection in Passive Lysis Buffer (Promega) and luciferase activity was measured using the Dual Luciferase Assay System (Promega) on a Synergy2 plate reader (Biotek). The level of Renilla luciferase activity was measured relative to firefly luciferase expressed from the same vector. These values were compared to the Renilla luciferase/firefly luciferase levels from a vector lacking either the Zeb2 or Zeb1 3′UTR. All values are relative to the mock treated cells.

### Immunoblot

Whole cell lysates were prepared using RIPA buffer (150 nM NaCl, 1% NP-40, 0.5% sodium deoxycholate, 0.1% SDS, 50 mM Tris pH 8.0 containing Complete Mini-protease Inhibitor Cocktail (Roche)). Protein concentration was determined using the BioRad DC protein assay kit (BioRad), and samples were resolved on 10% SDS-Page gels and transferred using a Transblot semi-dry transfer apparatus (BioRad). Blots were probed with antibodies to Zeb2 (kind gift of Anders Lund, University of Copenhagen), Cdh1, Cdh2 (BD Transduction), Vimentin (V5255, Sigma), and Zeb1 (AREB6) using ECL reagents (Pharmacia). α-tubulin and GAPDH were used as loading controls. All antibodies were used at a 1∶500 dilution.

### Cloning

The miR-141-200c cluster was amplified from genomic DNA purified using the DNeasy Blood and Tissue kit (Qiagen) by PCR using the HiFidelity PCR Master Mix (Roche), digested with BamHI and EcoRI and cloned into the pBabepuro retroviral vector. The miR-30 stem containing an shRNA against firefly luciferase was used as a negative control. The Zeb2 shRNA constructs were cloned into pll3.7 according to Rubinson et al. [58]. The primers used for the amplification of the miR-141-200c cluster are listed in Table S2. The miRNA control contains a luciferase shRNA cloned onto the stem of miR-30 [59], while the control shRNA targets firefly luciferase cloned as an shRNA. The primers used for cloning the shRNAs are listed in Table S2.

### Fluorescence microscopy

4TO7 and 4T1 cells were plated on glass cover slips and either left untreated or treated with miR-200b and/or miR-200c or a control miRNA mimic. 72 h post transfection the cover slips were washed extensively in phosphate buffered saline (PBS) and fixed with 2% paraformaldehyde in PBS. After additional washing, the cells were permeabilized with 0.2% Triton X-100 in PBS for 5 min. The cover slips were then washed and blocked with 10% FBS in PBS for 30 min. The cells were labeled with E-cadherin antibody (BD Transduction) in 10% FBS at RT for 2 h, washed extensively with 0.05% Triton X-100 in PBS and treated with Alexa488-conjugated donkey anti-mouse antibody (Molecular Probes) for 30 min. After further washing, the cover slips were mounted on glass slides with DAPI-containing Vectashield mounting media (Vector Laboratories) and images were acquired on an Axiovert 200 M microscope (Zeiss) using Slidebook Software (Intelligent Imaging Solutions).

### Soft agar assay

Tumor cells (5×10^3^) in complete medium containing 0.35% agar were overlaid on complete medium containing 0.8% agar in 6 well plates. The cells were grown for 10 days at 37°C plus 5% CO_2_. The number of colonies was determined by counting 5 fields of view from triplicate wells for each cell line.

### Thymidine incorporation

To measure cell proliferation, 4TO7 cells (5×10^5^ cells/well in 6-well plates) were seeded and after 24 h, transfected with miR-200c mimics (50 nM) or siRNAs targeting Zeb2 or luciferase using Lipofectamine 2000 (Invitrogen) following the manufacturer's protocol. After 48 h the cells in triplicate wells were incubated with ^3^H-thymidine (2 µCi/well) for 12 h and [^3^H]-incorporation was then measured using a liquid scintillation counter (Beckman).

### Transwell migration assay

Cells, harvested 48 h post transfection using 5 mM EDTA in PBS, were added (1.25×10^5^ cells/well) in serum free medium to triplicate wells of BD BioCoat™ Matrigel™ Invasion Chambers (BD Bioscience) and complete medium containing 10% FCS was added to the lower chamber. The invasion chambers were processed 24 h later as per the manufacturer's protocols, and migrated cells were stained using the Hematoxylin Stain Solution, Modified Harris Formulation (Amersco). Five random fields from each of the triplicate invasion assays were counted using phase contrast microscopy.

### Tumor implantation

12-week old female BALB/cJ mice (Taconic Farms) were injected subcutaneously into the right 4th mammary gland with indicated numbers of tumor cells in 100 µl PBS using a 30-gauge needle. Mice were palpated for tumor formation and tumor size was measured every 2 days by calipers. Tumor volumes calculated as Volume (mm^3^) = L×W^2^×0.4. Mice were sacrificed when the tumor size exceeded 15 mm in diameter in either direction or when the mice were moribund or at the end of the observation period (50 d). All animal experiments were approved by the Harvard University/Faculty of Arts and Sciences (HU/FAS) Standing Committee on the Use of Animals in Research and Teaching.

### Histology and immunohistochemistry

Primary tumors and whole lung, liver and brain tissues were dissected, fixed in 10% formalin (Sigma), embedded in paraffin, cut into 2 µm sections and stained with hematoxylin and eosin. For RNA isolation, mouse tissues were stored in RNAlater (Qiagen) prior to RNA extraction using RNeasy (Qiagen). PCNA staining in tissue sections was performed using a protocol of heat induced antigen epitope retrieval adapted from [60]. Briefly, paraffin was removed with xylene substitute and slides were rehydrated with decreasing concentrations of ethanol in distilled water. Slides were then immersed in 0.1 M citrate buffer, pH 6.0 (Lab Vision Citrate buffer for heat-induced epitope retrieval (ThermoFisher Scientific). The slide-containing staining jars were heated and allowed to boil for 2 min. The slides were kept in the boiled buffer for 20 min, before cooling at RT for 30 min. Slides were then blocked with PBS containing 5% normal donkey serum followed by blocking in avidin-biotin solution (Vector Laboratories). Endogenous peroxidase activity was quenched with 1% H_2_O_2_. Slides were then incubated overnight at 4°C with either polyclonal rabbit anti-PCNA antibody (Bethyl) or isotype-control rabbit IgG. Slides were washed and then incubated for 1 hr at RT with biotin goat anti-rabbit IgG (InnoGenex), followed by incubation for 40 min at RT with streptavidin-HRP (InnoGenex). The signal was developed using DAB solution according to the manufacturer's instructions (InnoGenex). Slides were then washed and mounted with SUB-X mounting medium (Surgipath). For E-cadherin staining, sections were digested with 2 µg/ml proteinase K (Ambion) at 37°C for 30 min and cooled in RT for 10 min. The slides were blocked with PBS containing 5% normal donkey serum followed by treatment with the avidin-biotin blocking Kit (Vector Laboratories). Endogenous peroxidase activity was quenched using 1% H_2_O_2_. The slides were washed and incubated with biotinylated goat anti-mouse Fab fragments (Sigma) for 1 hr at RT. Slides were then incubated with streptavidin-HRP for 40 min and the antigenic signal was amplified using the Tyramide Amplification Kit (Invitrogen) according to the manufacturer's instructions. Slides were then washed and mounted with Vectashield Mounting Medium containing DAPI (Vector Laboratories). Images were acquired and analyzed using Slidebook software (Intelligent Imaging) on a Zeiss Axiovert 200 M microscope.

## Supporting Information

Figure S1 The Zeb1 3′-UTR is a target of the miR-200 family of miRNAs. Cells were co-transfected with psiCheck2 vector that contains the full length Zeb1 3′-UTR and with miR-200b and/or miR-200c miRNA mimics. Renilla luciferase expression was normalized to firefly luciferase and the ratio then normalized to that of mock-transfected cells (*, p<0.0002).
(0.04 MB TIF)

Figure S2 PCNA staining of representative primary tumors and metastases from BALB/c mice. Tumors and metastases derived from implanted 4T1 cells or 4TO7 cells that were unmodified or infected with retroviruses expressing a control miR-30 stem insert or the miR-141-200c miRNA cluster within the miR-30 stem were stained with PCNA.
(0.89 MB TIF)

Table S1 miRNA expression profile of the mouse mammary tumor cell lines based on miRNA microarray analysis. miRNAs reporter names are referred to by their names in miRbase v9.0. Two biological samples for each tumor cell lines were used and two independent hybridizations (Trial 1 and Trial 2) were performed. The dynamic range for the microarray platform is over 5 logs. Background level is around 30 (arbitrary units) and averaged signal shown here is background subtracted and normalized. Detailed data analysis is described in the Materials and Methods Section. For miRNA candidate selection, averaged signal below 500 is considered as not detected.
(0.12 MB XLS)

Table S2 Primers and siRNA sequences.
(0.03 MB XLS)
