# Supplementary figures and images for: miR-200 Enhances Mouse Breast Cancer Cell Colonization to Form Distant Metastases
Source: PLoS One. 2009 Sep 29;4(9):e7181. doi: 10.1371/journal.pone.0007181 (PMC2749331; doi:10.1371/journal.pone.0007181)

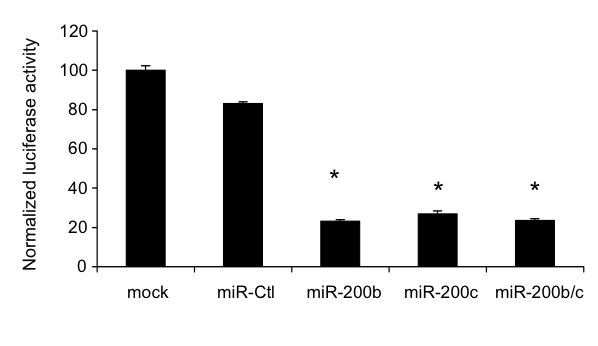

Supplement: Figure S1 — The Zeb1 3′-UTR is a target of the miR-200 family of miRNAs. Cells were co-transfected with psiCheck2 vector that contains the full length Zeb1 3′-UTR and with miR-200b and/or miR-200c miRNA mimics. Renilla luciferase expression was normalized to firefly luciferase and the ratio then normalized to that of mock-transfected cells (*, p<0.0002). (0.04 MB TIF) [file pone.0007181.s001.tif]

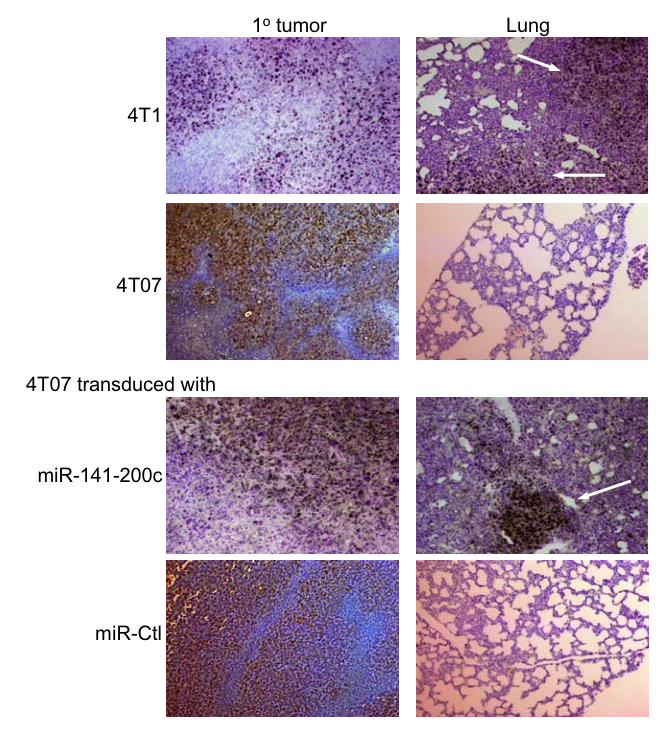

Supplement: Figure S2 — PCNA staining of representative primary tumors and metastases from BALB/c mice. Tumors and metastases derived from implanted 4T1 cells or 4TO7 cells that were unmodified or infected with retroviruses expressing a control miR-30 stem insert or the miR-141-200c miRNA cluster within the miR-30 stem were stained with PCNA. (0.89 MB TIF) [file pone.0007181.s002.tif]
